# Exploring pandemic preparedness through public perception and its impact on health service quality, attitudes, and healthcare image

**DOI:** 10.1038/s41598-025-95488-8

**Published:** 2025-05-20

**Authors:** Onur İzmir, Reda M. Lebcir, Oğuz Oypan

**Affiliations:** 1 Department of Management and Organization, Sinop University, Sinop, Turkey; 2 Hertfordshire Business School, University of Hertfordshire, College Lane, Hatfield, AL10 9AB UK; 3 Department of Wholesale and Retail, Antakya Vocational School, Hatay Mustafa Kemal University, 31060 Hatay, Turkey

**Keywords:** Perceived pandemic preparedness, Health service quality, Healthcare image, COVID-19, Türkiye, Health policy, Epidemiology, Health care economics, Influenza virus, Lifestyle modification, Population screening

## Abstract

Pandemic preparedness has gained increased significance in public health following the COVID-19 pandemic. Traditionally, it has been assessed from an internal organizational perspective, focusing on health sector readiness and addressing shortcomings. However, this perspective often overlooks the public’s perception of preparedness and its influence on behaviors within the healthcare sector. This study investigates how public views on pandemic preparedness shape attitudes, behaviors, and the image of the healthcare industry, which are critical in determining how individuals interact with health entities and respond to public health advice during a pandemic. To explore this, a set of hypotheses linking pandemic preparedness with health service quality, attitudes, and healthcare image was formulated. An online survey conducted in Türkiye gathered 322 responses. Hypothesis testing was performed using Structural Equation Modeling. The findings suggest that pandemic preparedness significantly impacts health service quality, public attitudes, and the image of the healthcare system during pandemic conditions. These results highlight the need to consider public perceptions of preparedness and their effects on behavior. Proactive communication strategies and public involvement in preparedness planning are essential for fostering a collective and informed response to the challenges posed by pandemics.

## Introduction

Although COVID-19 pandemic has negatively affected almost every economic and social sector in countries all over the world, the most affected sector by the pandemic, by a large margin, was healthcare^1,2^. Hospitals were quickly overwhelmed by COVID-19 patients stretching severely their resources and capacities leading to the closure of many services and interruption of care to patients with other conditions^3^. The pandemic showed that almost all countries were not adequately prepared to deal with a pandemic of this magnitude as illustrated by problems such as mask shortages, inadequate availability of ICU units and hospital beds, and limited stocks of critical drugs and pharmaceutical products^4,5^.

The global response to COVID-19 exposed significant weaknesses in healthcare systems worldwide, as confirmed by numerous studies. For example, a report by the World Health Organization (WHO), based on a survey from 105 countries looking at the impact of COVID-19 on health systems indicated that interruptions to health services occurred in almost every country^6^. Squalli^7^ analyzed data from over 140 countries in 2020 and found a positive association between COVID-19 mortality and factors such as aging populations, obesity rates, and healthcare expenditure, suggesting that even countries with substantial healthcare investments were not immune to the virus’s devastating impact. Therefore, there is a critical need to revisit the concept of “pandemic preparedness” as COVID-19 has exposed the vulnerabilities of health systems in both developed and developing nations.

Pandemic preparedness refers to planning activities taken in a country to reduce the transmission of the pandemic strain, decrease the number of cases, hospitalization and deaths, maintain essential services, and reduce the economic and social impact of the pandemic^8^. Traditionally, pandemic preparedness has been mainly evaluated through checklists, document analysis, and expert views^9–13^. This evaluation involves, generally, a situational analysis of a country focusing on the health system’s strengths and weaknesses to determine the degree of preparedness. Recommendations are then provided to policy makers to address the health system’s weaknesses and update preparedness plans to enhance the country’s ability to deal with pandemics. This pandemic preparedness perspective assesses the readiness of the healthcare sector and other public health entities to deal with pandemics, focusing on the performance of the *provider’s side* in relation to healthcare infrastructure, workforce training, and resource stockpiling^9,13–17^.

However, recent studies illustrate critical limitations of conventional provider-centric approaches to preparedness. For example, pandemic preparedness plans across 70 countries during COVID-19 showed that the majority included only their ministries of health during response, development, planning, and excluded all other stakeholders with communities being almost entirely left out of the process^12^. This approach to the development of preparedness plans neglects the involvement of key stakeholders and makes populations vulnerable during pandemics. Similarly, Villalobos et al.^13^ assessed pandemic preparedness and response plans from 35 Pan American Health Organization member states in the Americas region and identified many systemic problems. Only 14% of countries managed to address emergency coordination between different stakeholders and 80% omitted clear actionable guidance and communication strategies with the public.

This narrow view of pandemic preparedness characterized by the non-involvement of various stakeholders, the public, and vulnerable populations, who are key players in the success of implementing preparedness plans, constitutes a new challenge for public health authorities as they affect the behaviour and response of individuals during a pandemic^12^. In this context, Nunes et al.^18^ advocate the integration of behavioral elements into pandemic models to improve preparedness strategies and achieve better pandemic management decision-making. Based on the experiences gained during the COVID-19 pandemic, it can be concluded that human behavior had been a critical factor given the direct link between the observed risky behavioral patterns and the high transmission rates during the pandemic^18^.

The Theory of Planned Behavior (TPB), which posits that an individual’s adoption of a certain behavior strictly depends on attitudes and beliefs of the individual towards that behavior, offers a theoretical lens for this study. It enables the appreciation of the importance of attitudes, views, and perceptions in influencing and guiding behaviour. Individuals who have a positive attitudes and strong beliefs in a particular behavior or aspect are more likely to adopt, sustain, and act in line with it^19^. This aspect is important in the context of pandemic preparedness, as for plans to be implemented successfully, it is critical that people develop a favorable image of and trust in the healthcare sector and adopt positive beliefs and attitudes towards the policies and recommendations included in the plans. This is in line with past empirical studies underpinned by TPB, which showed that individuals with favourable attitudes towards pandemic preventive behaviors such as hand washing^20^, social distancing^21^, wearing masks^22^ and vaccine acceptance^3^ are more likely to engage in and even advocate for these behaviors.

The above findings reveal that, while the provider-centric approaches to pandemic preparedness focusing on resource allocation and institutional readiness are critical, it is equally important to bring broader societal and behavioral perspectives into preparedness frameworks to foster positive behavior, compliance, and therefore success. As such, action plans and activities related to pandemic preparedness need to be extended so that they can involve the public and positively shape its perception regarding the state of preparedness^2,23,24^. This will instill trust, promote adherence to public health guidelines, and enhance societal resilience during a pandemic^25,26^. This trust is important because pandemic control involves several measures directed towards the public such as lockdowns, social distancing, wearing of masks in public places, washing of hands, cleaning of homes and public spaces, following advice from medical staff, taking up vaccine, and so on^4,5^. The public perception of pandemic preparedness and confidence in the healthcare system are essential to ensure high public compliance with the measures^18,27^. Any mistrust, misinformation, and miscommunication can undermine public health efforts, leading to non-compliance and increased pandemic-related risky behavior^3^. The additional challenge here is that these behavioural aspects are not directly under the control of public health authorities, yet they determine whether preparedness plans translate into effective action during crises.

Despite the importance of these behavioral aspects for successful pandemic preparedness, it is surprising that past studies on pandemic preparedness gave little consideration to this dimension. The vast majority of research in the area of pandemic preparedness has focused on the internal preparations and planning activities undertaken by healthcare services and public health entities. This lack of understanding warrants further investigation given the importance of individuals’ perceptions and behaviours in facilitating the successful implementation of pandemic preparedness plans^18,26^. This indicates a research gap in pandemic preparedness studies as these predominantly adopt non-behavioral internal approaches focusing mainly on the managerial and preparation actions taken by health authorities.

To fill this gap, this study aims to understand the relationship between the degree of pandemic preparedness and individuals’ attitudes and behaviors, and their views of the healthcare sector. It also explores how preparedness activities benefit the healthcare sector and its performance. Given the significant effect of the COVID-19 pandemic on individuals and health systems worldwide, the psychological trauma it caused to the world population, and the need to learn from it to better prepare for the future, this pandemic has been selected as the focal subject in this research. The study took place in Türkiye, a medium income country, which was significantly affected by the pandemic^3^.

Türkiye’s experience provides a valuable case study, as it had over 107,000 confirmed cases by April 25, 2020, ranking among the most affected nations globally with an epidemic growth curve closely mirrored that of the U.S. and Italy during the early stages of the pandemic. (According to the latest shared data, Türkiye is ranked 11th with respect to the number of cases in the world. (worldometers.info).) This high number of COVID-19 cases significantly burdened its healthcare system^28^. Hospitals faced extreme pressure dealing with COVID-19 patients leading to the disruption of other healthcare services^28^. Furthermore, vaccine hesitancy further complicated the crisis, as low vaccine acceptance rate impacted public immunity^3^. Despite this, Türkiye managed to maintain a remarkably low case-fatality rate (2.51%), which was lower than that of most other highly impacted countries^29^ due to its strong healthcare infrastructure and capacity^30^. Even before the pandemic, Türkiye’s healthcare system was well-equipped (928 hospitals, 2840 emergency stations, 8000 family healthcare centers, 165,000 doctors, over 250,000 nurses, and 59,100 healthcare workers) and renowned for its high quality of care attracting patients from around the world for specialized treatments^31^. By examining the case of Türkiye, this study aims to understand how public trust and engagement influence the effectiveness of preparedness measures and to offer insights for improving future pandemic strategies via inclusion of the behavioral aspects.

The study is organized as follows: First, a review of the literature is conducted to develop the hypotheses, and this is followed by a description of methodology and the data collection methods. Next, the analyses of the measurement and the findings from the structural model are presented with a discussion of their implications. The last section covers the study’s conclusions.

## Material and methods

### Hypotheses development

#### Pandemic preparedness

The first guiding principles on pandemic preparedness were published by the WHO^32^. Few years later, the same organization highlighted certain concerns and dissatisfaction regarding global pandemic preparedness, which prompted the creation of a checklist for countries to assess and revise their pandemic preparedness plans^8^. The aims of these plans are to decrease transmission, number of cases and deaths, sustain health services during a pandemic, and balance the socio-economic costs of pandemics^9^. They involve the use of public health interventions such as vaccination campaigns to protect the public and minimize human transmission, and the provision of adequate medical care to the affected individuals.

The healthcare sector is central to pandemic preparedness planning, as it is responsible for providing care and treatment to individuals who become ill during a pandemic. Effective pandemic preparedness efforts enhance the capabilities of the healthcare sector and contribute to the development of healthcare staff knowledge and skills^17,23,24,32^. For instance, planning activities and training for large-scale vaccination programs—a key aspect of pandemic preparedness—have been shown to improve medical staff’s clinical abilities and health managers’ decision-making attributes, thereby positively influencing the quality-of-care provided^33^. In contrast, another research found that inadequate pandemic preparedness can result in stress, fatigue, and burnout among medical staff, which adversely affects the quality of care^23^. Similarly, it was reported that insufficient resources and lack of preparations significantly reduced the quality of care for COVID-19 patients in intensive care units^33^. The criticality and the importance of the link between pandemic preparedness and the quality of care offered by the health sector was also highlighted in another study^24^. Based on the above assertions, the following hypothesis is proposed:

##### H1

Pandemic preparedness level affects health services quality.

Pandemic preparedness affects also the perceptions and views of individuals and how they would be prepared to behave in response to pandemic prevention and control measures. This is known as attitude, which is defined as a person’s positive, negative, or neutral evaluation of a certain idea, object, or person, and the tendency to behave in a certain way in accordance with that evaluation^19^. It has been observed that when individuals perceive pandemic preparedness to be strong and adequate, they have more tendency to trust public health authorities and follow recommended pandemic protection measures^16,34^. Similarly, pandemic preparedness influences the interaction of the public with the healthcare sector given that pandemics tend to disrupt healthcare services. Strong pandemic preparedness signals to people that healthcare services are resilient even during a pandemic, which provides a feeling of trust (positive attitude) enabling individuals to continue seeking treatment in a normal way. Conversely, and as it was the case during the COVID-19 pandemic, if the public develops an impression that the healthcare sector is ill prepared to deal with the pandemic, unable to protect individuals from getting infected, and under-resourced to provide the required level of care for sick individuals, then this generates a deep level of worry and fear (negative attitude) causing a significant drop in the number of individuals seeking treatment^34^. These observations are in line with an emerging body of literature linking pandemic preparedness with attitudes towards the healthcare sector^18,33,35–37^. Consequently, the following hypothesis is suggested:

##### H2

Pandemic preparedness level affects attitudes towards the healthcare industry.

Countries with strong pandemic preparedness plans enjoy a positive image of their healthcare sector as highlighted by the WHO^10^. Given the complexity and multi-dimensional nature of preparations for pandemics and the variety of stakeholders involved in this process, a country with the ability to prepare and deal effectively with all the consequences of a pandemic is a testimony of the strength of its healthcare sector. If healthcare institutions are well-prepared for pandemics, it is very likely that this preparedness enhances their image and reputation within the society^36^. Conversely, it was observed that when the level of pandemic preparedness is inadequate, the capacity of healthcare facilities to cope with patients’ demand tend to decline due to the allocation of significant resources to deal with the pandemic^17,38^. Resource shortages and the resulting inability to address public care needs can erode confidence in the healthcare sector, affecting its overall image and reputation^37^. This finding was highlighted in a study in Nigeria, which found that the country’s health sector was underprepared for pandemics, and this negatively affected both the quality of care delivered to patients and the image formed by individuals’ regarding healthcare services^35^. Based on this, the following hypothesis is proposed:

##### H3

Pandemic preparedness level affects the healthcare image.

#### Health service quality

Service quality is defined as the degree to which a service meets the needs or expectations of customers^39,40^. This concept is particularly relevant across various sectors, including education, tourism, and banking. For example, it was observed that the quality of educational services significantly influences institutional image and student satisfaction^41^.In the hospitality and banking industries, high service quality is crucial for enhancing the reputation of hotels and financial institutions respectively, and influencing customer perceptions^42,43^.Given this, there is a relationship between the level of service quality provided to customers and the image they form of the service provider and the industry as a whole^44^. An organization that maintains a positive image due to high service quality may quickly lose this advantage if it fails to uphold service standards^45^, indicating the sensitivity of the relationship between service quality and industry image. This provides evidence of the importance of service quality in shaping the corporate image across various services sectors.

Within the healthcare industry, the relationship between service quality and healthcare sector image has been explored across various international settings and types of healthcare services. For example, this relationship was investigated in a general hospital setting, revealing that improved patient-perceived service quality positively influences brand image, word of mouth, and repurchase intentions^40^. In Indonesia, the impact of service quality and corporate social responsibility was examined on hospitals’ image finding that both factors significantly enhance hospitals’ image and value^46^.Another study examining the effects of service quality and facilities on patient satisfaction in a public health center in Indonesia showed that both service quality and facility conditions were crucial in shaping patient satisfaction and the center’s public image^47^. Similarly, Coutinho et al.^48^ assessed the influence of service quality and corporate image on patient satisfaction at a specialized cancer treatment facility in Brazil. The study demonstrated that, while service quality impacts patient satisfaction indirectly through the corporate image, the institution’s strong image significantly enhances patient perceptions. These studies collectively highlight that service quality is a critical determinant in forming a positive healthcare image across diverse healthcare settings, including general hospitals, specialized cancer treatment centers, and public health centers in various countries. Consequently, the following hypothesis is proposed:

##### H4

Health service quality affects the healthcare image.

#### Attitudes toward healthcare industry

Attitude, a multifaceted cognitive construct shaped by an individual’s beliefs and values, significantly influences human perceptions, behavior, and responses^49^. From a theoretical perspective, the Theory of Planned Behaviour^19^, which indicates that an individual’s adoption of a certain behavior strictly depends on attitudes and beliefs of the individual towards that behavior, provides a rationale in explaining the influence of attitudes and beliefs on behaviour and formation of image regarding people, ideas, and entities.

In the healthcare industry context, past literature has mostly focused on the effect of attitudinal factors such as perceived value, trust in healthcare providers, perceived quality of care, and health beliefs on patients’ satisfaction with healthcare services, patients’ loyalty to certain institutions, and their intention to seek medical care^50–54^. While these studies illustrate the importance of image in the formation of attitudes in the healthcare sector, they do not properly address the reverse relationship, that is how attitudes toward the healthcare sector influence its perceived image.

Recent research during the COVID-19 pandemic, however, provides some insights into the bidirectional nature of this relationship in the healthcare sector. For example, public attitudes toward nurses increased positively during the pandemic, as the public appreciated their extraordinary dedication and commitment, continuous visibility and perceived heroism, which directly enhanced the nursing profession’s image in Türkiye^55^.This study provides strong insights on how collective attitudes (e.g., gratitude, respect for nurses’ roles) can reshape the public image of healthcare professionals. Similarly, patient and caregiver attitudes toward telehealth and perceived COVID-19 risks changed interactions with and perceptions of healthcare institutions. Based on societal narratives, moral frameworks, and individual experiences, patients’ attitudes influenced the broader healthcare sector’s image^56^. In another study in Vietnam, it was found that the low confidence and trust in diagnosing and managing of Dementia lead to skepticism and lower image of the health sector^57^.

Within the general healthcare sector, it is interesting to note that most previous studies explored the impact of image on attitudes^53,58^. However, as healthcare is an intangible product, personal experiences and satisfaction (drivers of attitude) with the care and treatment provided to individuals significantly affect their views and formed image of the sector^59,60^.

The Healthcare industry provides pure services and hence shows distinct features from the goods industry. The services industry utilizes tangible goods to produce the (intangible) services, where mutual trust and commitment, experiences, narratives, and co-creation of value are important^61^. Therefore, healthcare’s intangible nature makes it uniquely susceptible to attitudinal shifts driven by personal experiences, societal narratives, and public trust. Positive attitudes such as confidence in providers, satisfaction with care can have a strong influence on the industry image as competent and patient-centric, while negative attitudes such as distrust, frustration with access barriers may potentially diminish its perceived value and reliability. Therefore, the following hypothesis is put forward:

##### H5

Attitudes towards the healthcare industry affect the healthcare sector image.

**Fig. 1:**
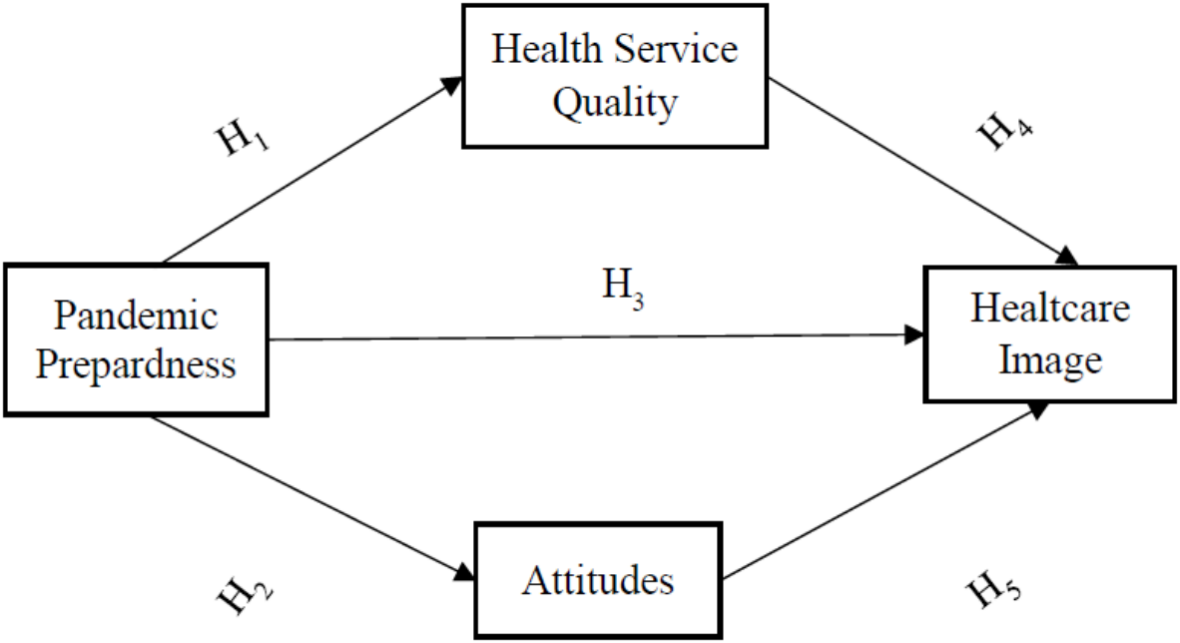
Model hypotheses.

The research model, in which the effect of perceived pandemic preparedness on health sector is investigated, is shown in Fig. 1.

### Methodology

A quantitative methodology based on data collected via a structured survey is selected for this study as this is appropriate to test the relationships between constructs^62^. The statistical technique selected to test the hypotheses in the model is the Structural Equation Modelling (SEM) technique. SEM is appropriate in situations where the same construct is an independent variable (cause) in some relationships and a dependent variable (effect) in others. For example, in the model presented in Fig. 1, the construct “Health Service Quality” is a dependent construct in hypothesis H1, and it is an independent construct in hypothesis H4. The use of SEM is appropriate to capture the nature and logic of relationships between key factors in a complicated context^62^.

Each construct was represented through a number of questions (items) in the survey. The constructs “Attitudes”, “Health Service Quality” and “Healthcare Image” items are derived from past published studies, whereas those representing the construct “Pandemic Preparedness” are developed as part of this research as there is no validated instrument in the literature to measure this construct. Prior to the application of SEM and in order to bring confidence in the representation of the constructs, the model was subjected to reliability and validity tests to ensure items comprehensively and validly represent the constructs they measure. These tests are essential to confirm the suitability of the SEM technique for the model.

#### Measures

The preparation of the survey for this study started by determining the items, which operationalize and measure the constructs in Fig. 1. The scale measuring the construct “Pandemic Preparedness (PP)” was fully developed as part of this research following a review of a sizeable body of literature^8–11,14,15,32,63–69^. This review indicated that PP refers to the perceived degree of efficiency in a pandemic outbreak with relation to the planning, coordination, communication, surveillance, control, and intervention efforts so as to (1) mitigate against the pandemic and its negative effects, (2) provide safety and security, (3) sustain economic, physical, and mental well-being of the public, and (4) expediate the normalization phase. Therefore, these concepts were included in the survey to represent the PP construct.

The “Attitudes (ATT)” construct is measured based on studies in the literature^70,71^. The construct includes such items as seeking treatment for oneself and others, saying positive things about and recommending hospitals, and intent and tendencies of individuals toward making health-related decisions under pandemic conditions. This construct reveals whether individuals will continue to seek treatment and make health-related decisions in a normal way if they feel that the pandemic is under control by public health authorities.

The “Health Service Quality (HSQ)” construct is measured based on the SERVQUAL scale designed to measure quality in the services industry^39,72,73^. The inclusion of the SERVQUAL scale items is motivated by Cronin and Taylor^74^, who suggested using these items for quantitative based studies. Service quality is represented in the scale by five dimensions namely: (1) tangibles (equipment, facilities), (2) reliability (ability to perform offered service accurately and reliably), (3) responsiveness (willingness to meet customers’ requests and provide quick service), (4) assurance (knowledge and kindness of employees and their ability to give confidence to customers), and (5) empathy (personal attention the organization shows to customers).

The items representing the “Healthcare Image (HCI)” construct are suggested by Kim et al.^75^ and adopted by Wu^58^. The image of the healthcare sector is expected to give impressions that the healthcare system is not disrupted during a pandemic and that the country is safe due to the policies taken to control pandemics, and the healthcare facilities in the country are renowned for excellence and high standards of care provision. The survey items were measured through a five-point Likert scale ranging from “strongly disagree” to “strongly agree”. The scales and items used in the survey are presented in Appendix 1.

#### Sample and data collection

The research population consisted of people over the age of 18 living in Türkiye and data was collected by conducting an online survey via Google Form. Participant consent was obtained and included in the online questionnaire process. At the start of the questionnaire, the purpose of the study was explicitly stated, and participants were informed that they have the right to withdraw from the study and not answer any question in the survey. Upon reading the study’s purpose and their rights, participants were able to proceed to the next section of the questionnaire either by clicking the “Next” button or exiting the questionnaire without responding to any question. The study was reviewed and approved by the Gümüşhane University Scientific Research and Publication Ethics Committee on 05/03/2023 (Decision No. 2023/2), confirming that all methods complied with relevant ethical standards, guidelines, and regulations. Informed consent was obtained from all participants prior to participation.

A total of 322 questionnaires were collected from which twenty-three (23) were deemed unusable for a number of reasons (missing responses, outlier issues etc.) leaving 299 usable questionnaires for the purpose of data analysis. Using the Gpower tool, a post hoc analysis was conducted to compute the achieved power of the sample. In the research model, the outcome variable with the highest number of predictor variables (Healthcare Image) had three predictors (Pandemic Preparedness, Attitudes, Health Service Quality). Cohen’s^76^ effect size (f^2^) was calculated as high for the model (0.37). The statistical power analysis conducted by utilizing Gpower, achieved a power value of over 95%, indicating that the sample size was sufficient to test the hypotheses in the research model without the risk of falling into type II error.

In addition, an a priori power analysis was also performed. According to the output of the analysis, to achieve 95% power with a high Cohen’s^76^ effect size (0.35), it is determined that minimum sample size should be no less than 54 participants to test the research model with the minimum risk of Type II errors. These results confirm that the sample size of 299 participants is well above the minimum threshold value to perform robust statistical analysis to identify the significant effects in the research model. Regarding the sampling process, the aim was to achieve diversity in the sample in terms of age, education, and location to reflect the population of Türkiye and to reduce the likelihood of extreme bias. Furthermore, the design of this study was made for hypothesis testing, specifically examining the behavioral outcomes of perceived pandemic preparedness, rather than concluding with population-level inferences and generalisations.

## Results

### Evaluation of the measurement and structural model

The model was subjected to several tests to ensure that it is fit for the application of SEM. First, we checked construct reliability and all constructs in the measurement model had a Cronbach-α score higher than 0.7 confirming reliability (See Appendix 2). Next, tests regarding construct validity, which includes convergent validity and discriminant validity were conducted. Convergent validity is achieved if items loading for standardized items are higher than 0.7 and statistically significant^77^, the Average Variance Extracted (AVE) is greater than 0.50, and Composite Reliability (CR) exceeds 0.70^77,78^. However, they also note that loadings of 0.5 or higher can be acceptable, especially in exploratory research or when developing new scales, as long as the AVE and CR meet the required thresholds. For discriminant validity, the condition is that the square root of a construct’s AVE should be greater than its correlations with other constructs in the measurement model^79^.

**Table 1:** Construct validity of the measurement model.

Constructs	CR	AVE	ATT	PP	HCI	HSQ
ATT	0.960	0.859	**0.927**			
PP	0.928	0.544	0.588	**0.737**		
HCI	0.934	0.704	0.796	0.712	**0.839**	
HSQ	0.958	0.820	0.596	0.575	0.746	**0.906**

PP, Pandemic Preparedness; ATT, Attitudes; HCI, Healthcare Image; HSQ, Health Service Quality; CR, Composite Reliability; AVE, Average Variance Extracted.

Factor loading values indicate that all items, except for four (PPP10, PPP11, HSQ13, HCI5), are greater than 0.7 (See Appendix 2). However, as the factor loading for these four items is above 0.5, this is considered acceptable according to Hair et al.^77^, indicating strong reliability and validity. Results in Table 1 show that the lowest CR for all constructs is 0.928 and the lowest AVE is 0.544, meaning that the convergent validity criteria are met. The square root of AVE of each construct in the measurement model (Bold in Table 1) is greater than the correlations among the constructs in the measurement model indicating that discriminant validity of the constructs in the measurement model is also achieved. Therefore, the SEM method is applicable for the model in Fig. 1.

In this study, x^2^/df (a chi-square/degrees of freedom ratio), CFI (Comparative Fit Index), RMSEA (Root Mean Square Error of Approximation) and SRMR (Standardized Root Mean Square Residual) are used to test the model fit to the data^77,80^. The measurement model was found to exhibit good fit indices, with a x^2^/df of 2.321, CFI of 0.919, RMSEA of 0.067, and SRMR of 0.052. These results provide additional evidence that the SEM technique is applicable to the model.

### Statistical analysis findings

According to the SEM path analysis results in Table 2, the relationships in the model are significant and all hypotheses are supported. Pandemic preparedness positively affects health service quality (β = 0.575, *p* = 0.003), attitudes (β = 0.588, *p* = 0.002), and healthcare image (β = 0.259, *p* = 0.001) confirming that hypotheses H_1_, H_2_, and H_3_ are supported. Similarly, health service quality and attitudes are significant predictors of the healthcare image as hypothesis H_4_ (β = 0.329, *p* = 0.003) and H_5_ (β = 0.448, *p* = 0.002) are also supported.

**Table 2:** Results of the SEM statistical analysis.

Hypothesis	Supported	Path coefficient (β)	*p* value	R-square
H1 (PP-HSQ)	Yes	0.575	0.003	0.331
H2 (PP-ATT)	Yes	0.588	0.002	0.346
H3 (PP-HCI)	Yes	0.259	0.001	0.786
H4 (HSQ-HCI)	Yes	0.329	0.003	0.786
H5 (ATT-HCI)	Yes	0.448	0.002	0.786

The values of R^2^ for the constructs “Health Service Quality”, “Attitudes”, and “Healthcare Image” are 0.331, 0.346 and 0.786, respectively. The R^2^ figures represent the variability of the dependent construct explained by the independent constructs in the hypotheses involving the dependent construct. For example, 78.6% of the variability in the dependent construct “Healthcare Image” is explained by the independent constructs: “Pandemic Preparedness” (Hypothesis H3), “Health Service Quality” (Hypothesis H4), and “Attitudes” (Hypothesis H5).

Although “Pandemic Preparedness” is the only antecedent construct for both “Health Service Quality” and “Attitudes”, the variance for these two constructs explained by pandemic preparedness is satisfactory (33.1% and 34.6% for the constructs Health Service Quality and Attitudes, respectively). The three constructs predicting healthcare image (pandemic preparedness, health service quality, attitudes) explain a considerable amount of its variance (78.6%). In summary, these results reveal that the level of pandemic preparedness in a country plays a vital role in enabling the healthcare sector to provide adequate healthcare services quality to patients, influences individuals’ attitudes, and provides a positive image of the health sector in the country.

The model in Fig. 1 shows that the construct “Healthcare Image” is affected directly and indirectly by the construct “Pandemic Preparedness”. The direct relationship between these two constructs is represented in hypothesis H3 whereas the indirect relationships involve the construct “Health Quality Service” (Hypothesis H1 then H4) and the construct “Attitudes” (hypotheses H2 then H5). Therefore, we explored these indirect effects to provide a deeper understanding of how pandemic preparedness influences healthcare image. The standardized total effect composed of both the direct (pandemic preparedness to healthcare image) and the indirect (pandemic preparedness to healthcare image via health service quality and attitudes) is 0.712 (*p* < 0.01). For the indirect effect only, the results indicate that the effect of pandemic preparedness on healthcare image through the mediation of both attitudes and health service quality is 0.452 (*p* < 0.01).

Having established the existence of indirect effects influencing the relationship between the constructs “Pandemic Preparedness” and “Healthcare Image”, we carried out a further analysis to determine the relative strengths of the two mediating constructs “Health Service Quality” and “Attitudes” on this relationship. The results indicate that the indirect effect of pandemic preparedness on healthcare image through the mediation of health service quality is significant at *p* < 0.001, and the magnitude of this mediation effect is 0.173. Regarding the mediation effect through attitudes, the magnitude is 0.240 and the mediation is significant at *p* < 0.001. This means that the relative mediation effect of attitudes is stronger than that of health service quality.

To conclude, pandemic preparedness of a country directly and indirectly shapes its healthcare image. In addition, attitudes towards the health sector, and the level of health service quality provided during a pandemic play significant roles in the development of a good image of the healthcare industry at a country level.

## Discussion

Historically, research in the area of pandemic preparedness has primarily focused on the issues centered around public health policy and governance, operational and managerial preparations to control pandemics, and delivery of health services from an internal healthcare stakeholders’ perspective^9,11–13^.However, COVID-19 has had a profound effect on individuals, societies, and states reminding everyone of the importance of having robust preparedness plans to deal with pandemics and to make the public aware that the healthcare sector is ready when a country is hit by such events^18,24,27,81^. In addition, it is important to understand the public perception of pandemic preparedness as this can illuminate the ways in which such perceptions influence individual attitudes, behaviors, and the mental constructs individuals form of the healthcare sector. These factors can profoundly impact the effectiveness of pandemic control measures and the quality of care provided to patients. This study focuses on this aspect by examining pandemic preparedness from a behavioral perspective, as the spread of infectious diseases and the effectiveness of public health interventions depends, to a great extent, on human behavior.

The dominant perspective in pandemic preparedness is still internally driven as it focuses on the health sector activities such as stockpiling of vaccines and personal protective equipment, training of healthcare workers, building of ICU and hospital beds capacity, and other aspects of logistics and supply chain management^13,17^. However, these efforts neglect the noncompliant and negligent public behaviors, which causes high transmission rates and resistance to vaccine uptake. Stockpiling vaccines matters little if hesitancy undermines uptake^3^ and high ICU and hospital beds capacity becomes irrelevant if distrust delays care-seeking^18^. Therefore, as an alternative to the internal stakeholder approaches, this study proposes an external behavioral perspective centered around individual perception of pandemic preparedness and its effect on their behaviour and the image they form on the healthcare sector.

The findings of this study indicate that the pandemic preparedness level of a country positively affects health service quality. This is in line with the findings of Qari et al.^15^ and De Micco et al.^82^ that the development of strong pandemic preparedness plans sharpens medical staff knowledge and skills and enhances health managers’ problem solving and decision making. This, in turn, increases the ability of healthcare services to deliver better quality of care to patients. Similarly, high vaccination coverage of health staff, a critical element of pandemic preparedness, provides staff with the protection they need enabling healthcare organizations to increase their capacity and resilience, improving quality of care^72^. Similarly, Oppenheim et al.^69^ claim that not being ready for pandemics affects the normal functioning of health services negatively impacting quality of care and pandemic risk. Therefore, high health service quality may serve as a strong indicator of the robustness of both pandemic control measures and the healthcare system enhancing public confidence. This stimulates timely care-seeking behavior and compliance with preventive strategies, thereby helping to curb disease progression, increased mortality rates, and the exacerbation of non-pandemic-related illnesses^18,27^. Therefore, this link between pandemic preparedness and health services quality is an important aspect, which health policy makers should pay attention to when making decisions regarding, for example, the level of spending on pandemic preparations plans and activities.

Pandemic preparedness plays an important role in shaping attitudes of the people towards the healthcare sector. This is consistent with recent studies that emphasized the relationship between pandemic preparedness and attitudes^4,5,83,84^. For example, Ashcroft et al.^83^ found that when medical students are involved in pandemic preparedness activities, they tend to develop a positive attitude toward the healthcare sector’s capacity to effectively manage pandemics. This, in turn, enhances their level of readiness and commitment to fulfilling their responsibilities when required during a pandemic. In addition to healthcare staff, the perceptions of pandemic preparedness can also influence the attitudes of the general public. Health services are usually disrupted during pandemics causing negative attitudes toward the healthcare sector^1^. This could lead individuals to undesirable attitudes and behaviour such as avoiding visiting healthcare facilities to seek treatment and spreading negative claims about the ability of the health sector to provide adequate care causing others to adopt the same attitude. Therefore, it is important that policy makers appreciate the importance of the link between pandemic preparedness and attitudes and behaviors of the public, and how this impacts the health and wellbeing of the public during a pandemic^34^.

The existence of a positive link between pandemic preparedness and the image of the healthcare sector of a country is an important outcome of this study. When a country is perceived to be well prepared to deal with pandemics, people tend to attribute this adequate preparedness to the strength of the healthcare industry and will believe that the health system has the necessary resources and the clinical and management expertise to cope with the pressures and demand put upon it by the pandemic. This would lead individuals to have confidence in and form a positive view of the healthcare sector. Conversely, if the level of pandemic preparedness is inadequate, this can have serious implications for the health sector and the healthcare image of a country. For example, WHO^10^ emphasizes that pandemics can disrupt the social order, threaten the provision of essential public services, and hinder the production and distribution of goods and services to the population. Although the main causes for these distributions lie within the organizations responsible for public services, economic, and supply chain activities, which are outside of the healthcare sector, they are generally perceived by the public as being caused by the inability of the health sector to effectively control the spread of the pandemic and provide the required care to the public. Consequently, a negative view of the healthcare sector is formed as it is seen to be the main cause of these disruptions. Based on a recent study, the integration of AI-based technologies into operations can help overcom barriers in the supply chain^85^. By incorporating these technologies into healthcare operations, the barriers can be properly addressed, and hence pandemic-related challenges can be managed more efficiently and effectively. Therefore, the ability of health policy makers to robustly plan and respond to pandemics can significantly mitigate against these risks, alleviate the public’s fear, and provide assurances that the pandemic is dealt with effectively^37,86^. This enhances the image of a country and its healthcare sector reducing the negative effects of the pandemic on other sectors such as tourism, transport and aviation, manufacturing, and education, hence stimulating and keeping the economy going during the difficult times of the pandemic^66^. Therefore, the findings of this study imply that the broader behavioral impacts of perceived pandemic preparedness may not only remain limited to the healthcare industry and have the potential to extend to other industries.

The level of the health service quality is also an important antecedent of a country’s healthcare image. This is consistent with previous studies supporting the effect of health service quality on hospital and country’s healthcare image^87,88^. It is known that sustaining a high level of health service quality during a pandemic is challenging given the disruptions it causes to the healthcare sector, the high number of infected individuals, the pressure on medical and public health infrastructures, and the excessive workload of healthcare personnel ^89^. However, the ability of a country’s health sector in implementing strong pandemic preparedness plans can counteract the negative effects of pandemics. Such measures enable healthcare services to continue providing high quality medical care to all in need, meeting the expectations of the public. This instills public satisfaction and confidence that the health sector can deliver excellent services during challenging times, thereby enhancing the image of the country’s healthcare sector^36,86^.

Attitudes toward healthcare are positively associated with the image of the healthcare sector. Individuals who hold favorable attitudes toward the state of the healthcare sector are more likely to form a positive perception of it. A series of positive experiences with healthcare services, which shape these attitudes, contribute to the view that the healthcare industry is robust and reliable. This, in turn, fosters the formation of a positive image of the sector. Therefore, it is crucial to strengthen health systems to maintain individuals’ positive attitudes and build their confidence in the sector’s ability to protect their health and well-being^18,27^.

A key implication of this study is that pandemic preparedness should go beyond the planning processes and preparation activities carried out by the healthcare sector and other entities in charge of managing pandemics. Pandemic preparedness activities should also include clear and effective communication with the public regarding both the expected role of individuals during the pandemic and the level and state of pandemic preparedness in the country. Informing individuals about their role involves several aspects such as the precautionary measures to adopt, adherence to public health prevention policies, guidance on seeking help during the pandemic, and information related to the availability and effectiveness of treatments and vaccination programs. Clear and effective communication is of critical importance given the negative impact of misinformation during pandemics as evidenced during the recent COVID-19 crisis^3^.

It is essential to keep the public informed about the extent of pandemic preparedness and the capacity and resources to be utilized by the healthcare sector during a pandemic. Baccega et al.^90^ emphasize the importance of accurate epidemic forecasting in fostering public awareness and participation in disease control efforts. Effective communication of these forecasts can significantly enhance public trust and compliance with pandemic preparedness measures. This communication serves to reassure the public that their safety will be ensured and that they will receive the necessary care. This, in turn, positively influences the perception and behavior of individuals during the pandemic and contributes to greater adherence to public health protocols, mitigates the negative influence of misinformation, and encourages people to continue seeking medical advice and treatment without hesitation. Proactively addressing concerns and dispelling fears can help overcome situations where members of the public deliberately avoid visiting clinics and hospitals to receive treatment as observed during the COVID-19 pandemic. This avoidance mostly resulted from the fear of catching the virus and the perception that the healthcare sector was not well-prepared to manage the pandemic as medical staff were completely overwhelmed by the high number of COVID-19 patients^34^.

The mediating effect of attitudes on the relationship between pandemic preparedness and the healthcare sector’s image is an important finding of this study. Given the importance of a country’s healthcare image and its dependence on public attitudes, it is important that healthcare policy makers broaden their focus beyond the internal preparations to inform and involve the public. Perceptions of the degree of pandemic preparedness play a critical role in shaping public attitudes and behavior during a pandemic. Therefore, it is crucial to keep the public fully informed about the plans, preparations, resources, and capabilities that will be mobilized in response to a pandemic. In addition, it is equally important to explain the roles and the responsibilities of individuals during a pandemic, as active public engagement and cooperation during a pandemic are strongly associated with better outcomes in terms of controlling and reducing the negative impact of pandemics^91^. The use of modern media and communication tools is imperative in facilitating and enabling a smooth information sharing process. Investing in and effectively using communication tools should become a standard element of pandemic preparedness to provide information to the public before and during a pandemic. When distrust towards the healthcare system and public authorities prevails in a country, individuals may delay or avoid seeking care^27^. If this pattern of behavior spreads to the general population, it may exacerbate the progression of disease to more severe stages. Therefore, the negative perception of the pandemic preparedness has the potential to result in increased complications and mortality, and ultimately, a greater strain on healthcare resources as untreated or undiagnosed cases can, further, contribute to ongoing community transmission of the virus, making it more difficult for public health efforts to control the pandemic^18^.

Given the beneficial consequences of individuals developing a positive image of the healthcare sector, it is vital to actively engage the public throughout the planning and implementation of the policies and measures to control and manage the pandemics^25,26^. This approach helps maintain public confidence in the robustness of the pandemic preparedness plans and the readiness of the public health authorities to combat the pandemic.

Although the data was collected during the later stages of the pandemic, the findings still represent the perceptions of the public regarding the impact of the pandemic and the preparedness to deal with it. The unprecedented scale, global scope, and strong social and economic impacts of the COVID-19 pandemic have led to significant concerns by populations and negatively affected their mental health for an extended period of time^92,93^. In addition, significant prevalence of long COVID-19, where patients continued to suffer from its symptoms for a significant period following the initial outbreakhas further prolonged public awareness of the pandemic’s seriousness and threat. For example, Egypt, a country geographically and demographically close to Türkiye, has experienced a long pandemic prevalence rate of 86%^94^. This phenomenon likely contributed to sustained public anxiety, as evidenced by the high prevalence of depression and anxiety in Turkish society during the pandemic^95^. This is why our finding regarding the positive image of the healthcare sector and its ability to effectively cope with COVID-19 is not considered to be very different from the early stages of the pandemic^96^.

It is important to note that this study focused on public perceptions of pandemic preparedness and its effect on individuals’ behavior and views of the healthcare sector. As such, it does not evaluate the country’s actual level of preparedness and the suitability of the plans to combat epidemics. While most countries had existing plans based on past outbreaks such as SARS or Ebola, these were inadequate to address the unprecedented scale of the COVID-19 pandemic. It is encouraging that lessons are being learned from this crisis to develop stronger and more robust plans to deal with future epidemics^97^ including the need to keep the public informed about national preparedness and response efforts.

## Conclusion

Pandemic preparedness is not a new topic, but it has gained renewed emphasis following the profound and multi-dimensional impact of COVID-19 on individuals, countries, and health systems. In this study, while recognizing the importance of the traditional internal perspective on pandemic preparedness focusing on institutional readiness, resource allocation, healthcare infrastructure, and policy-driven response mechanisms essential for effective crisis management^13,17^, this study furher enhances the body of knowledge in the pandemic preparedness literature by highlighing the critical role of public perceptions and engagement in shaping the overall effectiveness of preparedness efforts. This external perspective, covering broader implications of perceived pandemic preparedness for the healthcare sector, introduces the behavioral dimension to the field of pandemic preparedness. This new dimension can be considered as a complementary approach to the classical research in this field, which has historically focused on the internal preparation and planning activities carried out by public health entities.

One of the most important conclusions of this study is that pandemic preparedness is a dual-faceted concept covering: (1) the internal preparation activities managed by countries’ public health entities, and (2) the external views and perceptions of individuals regarding the level of preparedness. While these two perspectives are interrelated, they may not always align, especially when public communication and participation in preparedness and planning activities are absent or inadequate, as individual views may deviate from the actual state of preparedness. This misalignment is critical because if the public perceives that a pandemic is not being effectively prepared for and managed, it may lead to non-compliance behavior with health recommendations resulting in adverse outcomes^18,66^. Conversely, an overly optimistic perception of preparedness compared to the actual level of readiness may also create a false sense of security, fostering complacency, risky behavior, and further non-compliance behaviour among individuals.

Therefore, a key finding of this study is that developing robust pandemic preparedness plans, while essential, is not sufficient to ensure public cooperation, a prerequisite for the successful implementation of the plans. Effective communication about the robustness and adequacy of the preparedness plans and, conveying this message clearly to the public, is just as critical as the development of the plans themselves. The COVID-19 pandemic demonstrated that public adherence to unprecedented health measures was instrumental in transforming preparedness plans into effective actions during the pandemics. These findings send an important message to policy makers, as there is a clear disparity between the theoretical aspects and operational realities in pandemic preparedness frameworks.

This study demonstrated that pandemic preparedness may have advantages to the healthcare sector beyond the management of pandemics. Preparedness activities have transferable benefits in terms of improving knowledge and skills of healthcare staff, fostering better decision-making processes among policy makers, and enhancing the analytical and coordination capabilities of various stakeholders involved in pandemic control^98^. These benefits can lead to a positive impact on the healthcare sector at large, extending beyond the lifecycle of the pandemic and the specific entities directly involved in pandemic response.

There are certain limitations that need to be acknowledged in this study. First, quantitative research methods were used, therefore, the findings are limited to the variables included in the model. Future research can explore the contribution these to explain behavioral effects of perceived pandemic preparedness and additional variables using qualitative research methods to gain deeper insights. Second, the effects of pandemic preparedness were mainly examined with a focus on the healthcare sector. However, pandemic preparedness is of great importance not only to the health sector, but also for numerous other sectors and stakeholders, warranting further investigation in future studies. Third, although due care was given to the inclusion of characteristics representative of the Turkish population, the results of this study may not be generalizable to the entire population of the country. Future studies can select more representative samples to test the generalizability of the findings to the whole population. Finally, the scale measuring pandemic preparedness in this study was developed based on the findings of previous literature and there is a need for future studies to confirm the extent of the validity of the scale.

### Theoretical contributions

This study investigates the concept of pandemic preparedness from a behavioral perspective, which is a departure from the traditional, internal/provider-centric, perspective. As such, it contributes to the literature by emphasizing the role of perceived pandemic preparedness in shaping the effectiveness of pandemic response measures through public attitudes, behaviors, and overall trust in healthcare systems.

This study confirms the theoretical underpinnings of beliefs-perceptions-attitudes driven decision-making processas suggested by Fishbein and Ajzen^99^ and Ajzen^19^ in TPB. The findings suggest that pandemic preparedness is a concept with two facets, including both the actual preparedness of a country (as measured by internal metrics) and the perceived preparedness by the public. In this context, actual pandemic preparedness provides insights into the preparedness level of the country in terms of resources and infrastructure whereas perceived pandemic preparedness reflects the extent to which the society perceives the country to be ready for a pandemic. This distinction is crucial because, as Fishbein and Ajzen^105^ and Ajzen^19^ have argued, individuals act based on their beliefs, perceptions, and attitudes. Therefore, even if a country is actually well-prepared from the traditional perspective in terms of its resources and infrastructure, public behavior during a pandemic may still deviate from the desired behaviour if the perceived preparedness was deemed inadequate by the public. The discrepancy between objective (institutional) and perceived (public) components of pandemic preparedness can lead to non-compliance with public health measures, vaccine hesitancy, and delayed care-seeking behavior, ultimately undermining the effectiveness of pandemic control strategies.

The above-mentioned theoretical implications are important for a successful pandemic control action plan. The relationship between beliefs, perceptions, attitudes, and behaviour advocated in TPB offers an important frame to explain drivers of behavior and their implications. If members of the society believe that the country has the required capabilities to control the pandemic, their response will be positive, reducing transmission risks and leading to better pandemic related outcomes. Conversely, if individuals have a pessimistic view of the healthcare sector’s readiness to manage the pandemic, they may be less willing to fulfill their duties and more likely to engage in risky behaviours, worsening the crisis. Therefore, one of the most important theoretical implications of this study is the fact that developing robust and flawless pandemic preparedness plans is not sufficient to influence behaviors of the individuals during a pandemic outbreak. Communication regarding the robustness of pandemic preparedness plan and persuasion of individuals of the strength of the health sector and the merit of compliance with public health authorities’ directives are required to ensure positive behavior of the society and outcomes during the pandemic.

### Practical implications

The practical implications of this study offer important insights for policymakers, healthcare administrators, and public health officials. First, because perceived pandemic preparedness has broader behavioral implications, there is a need for the design of clear, transparent, and proactive communication strategies with the public regarding pandemic preparedness plans. The gap between actual preparedness and public perception can be bridged through effective communication strategies, which instills trust in healthcare institutions and encourages compliance with public health measures. Social media, mobile apps, and various related digital platforms as suggested by Kordestani et al.^3^ can be important tools during the dissemination of information to the public about the latest pandemic developments, preventive measures, and available resources. Policymakers can utilize these tools to overcome misinformation and enhance public awareness and engagement to achieve effective pandemic response.

Second, this study highlights the importance of involving various stakeholders, among which the public is the most important, in pandemic preparedness planning. The acceptance of preparedness measures can be enhanced by the inclusion of value co-creation approaches through public participation and social marketing efforts as individuals can be more willing to adopt and advocate the rationale behind the guidelines that have been collaboratively developed. Furthermore, the participation of the public can make preparedness plans more equitable and inclusive by especially identifying and addressing the concerns of vulnerable populations.

Third, as part of pandemic preparedness planning, possible scenarios on behavioral barriers to pandemic control should be discussed and addressed. Vaccine hesitancy, reluctance to seek timely medical care, and resistance to non-pharmaceutical interventions (e.g., masks, social distancing) are influenced by public trust and perception of preparedness. Any negative behavior affecting compliance and increasing risks can cause serious public health problems and threaten the overall well-being of the population. Therefore, incorporating behavioral insights into pandemic epidemiological models and preparedness plans can improve intervention strategies, and contribute to better public health outcomes.

Fourth, maintaining high-quality healthcare services during a pandemic, whenever possible, is essential for preserving public trust and confidence in the healthcare system. Efforts to optimize service operations during the pandemic can bear fruitful results and resonate with the public. Increased service quality perception coupled with positive attitudes toward behavior in the healthcare sector can result in the formation of an enhanced image of the healthcare industry. By ensuring that healthcare services remain accessible and are able to provide high quality of care during a crisis, policymakers can mitigate against the negative impact of pandemics on public health and individuals’ behaviour.

In conclusion, while recognizing the importance of traditional pandemic preparedness approaches, this study adds a new dimension by highlighting the significance of a more inclusive and public communication-focused approach to pandemic preparedness. By properly addressing both the internal and external dimensions of preparedness, healthcare authorities can manage effective response, coordination, and control future pandemics.

## Data Availability

Data is available upon reasonable request from corresponding author.

## Appendix 1

### Perceived pandemic preparedness^8–11,14,15,32,63–69^

1. In general, I think my country is prepared for the COVID-19 outbreak (PP1).
2. I think that communication channels are transparent and open in terms of information sharing about the pandemic (PP2).
3. Government bodies are successfully managing the pandemic (PPP3).
4. I think that the quarantine applications are carried out successfully (PPP4).
5. There are no serious disruptions in the functioning of the health sector (PPP5).
6. There is no shortage of equipment necessary for the detection of the disease (PPP6).
7. COVID-19 patients are treated with care using all available treatment options (PPP7).
8. Science committee makes timely and appropriate interventions during the pandemic (PPP8).
9. There is no capacity or personnel shortages at the hospitals (PPP9).
10. Economic precautions taken for the pandemic are sufficient (PPP10).
11. There is no disruption in the supply of the basic needs such as food, electricity, water, natural gas, internet etc. (PPP11).

### Health service quality^39,73,74^

#### Tangibles

1. The hospitals in Türkiye have up-to-date equipment (HSQ1).
2. The physical facilities of the hospitals in Türkiye are visually appealing (HSQ2).
3. The health professionals of the hospitals in Türkiye are well dressed and appear neat (HSQ3).
4. The materials and equipment used during the treatment process are of good quality (HSQ4).

#### Reliability

5. The hospitals in Türkiye do what is required to be fulfilled during the treatment process by the time they promised (HSQ5).
6. When their patients have a problem, the hospitals in Türkiye show a sincere interest in solving it (HSQ6).
7. The hospitals in Türkiye fulfill the treatment of the illness right the first time (HSQ7).
8. The hospitals in Türkiye provide the treatment at the time they promised to do so (HSQ8).
9. The hospitals in Türkiye keep error-free records of the information related to their patients (HSQ9).

#### Responsiveness

10. The health professionals of the hospitals in Türkiye tell patients exactly all the required dates and hours of the appointments in the treatment process (HSQ10).
11. The health professionals of the hospitals in Türkiye provide required health services to the patients promptly (HSQ11).
12. The health professionals of the hospitals in Türkiye are always willing to help the patients (HSQ12).
13. The health professionals of the hospitals in Türkiye are never too busy to respond the requests of the patients (HSQ13).

#### Assurance

14. The behaviors of the health professionals of the hospitals in Türkiye instill confidence to the patients (HSQ14).
15. The patients in the treatment process feel safe at the hospitals in Türkiye (HSQ15).
16. The health professionals of the hospitals in Türkiye are consistently courteous with the patients (HSQ16).
17. The health professionals of the hospitals in Türkiye have the knowledge to give satisfactory answers to patient questions (HSQ17).

#### Empathy

18. The hospitals in Türkiye give patients individual attention (HSQ18).
19. The hospitals in Türkiye have operating hours convenient to all patients (HSQ19).
20. The hospitals in Türkiye have healthcare professionals who give the patients personal attention (HSQ20).
21. Hospitals in Türkiye provide you with the best treatment you want to receive (HSQ21).
22. The healthcare professionals of the hospitals in Türkiye understand the specific needs of the patients (HSQ22).

### Attitudes^70,71^

1. I would like to get treatment in Türkiye for myself (ATT1).
2. I would tell people positive things about the hospitals in Türkiye (ATT2).
3. I would like my family and friends to get treatment in Türkiye (ATT3).
4. I would suggest the hospitals in Türkiye to the people around me (ATT4).

### Healthcare image^58,75^

1. The hospitals in Türkiye have a good reputation (HCI1)
2. The hospitals in Türkiye have excellent facilities (HCI2).
3. The hospitals in Türkiye have a comfortable environment (HCI3).
4. The hospitals in Türkiye provide a sense of trust (HCI4).
5. The doctors in Türkiye treat patients well (HCI5).
6. The hospitals in Türkiye have the most advanced medical equipment (HCI6).

## Appendix 2

### Perceived pandemic preparedness

**Table:**

Items	Factor loading	Cronbach α
PPP1	0.784	0.925
PPP2	0.783	
PPP3	0.791	
PPP4	0.756	
PPP5	0.793	
PPP6	0.773	
PPP7	0.749	
PPP8	0.729	
PPP9	0.710	
PPP10	0.681	
PPP11	0.521	

### Health service quality

#### Tangibles

**Table:**

Items	Factor Loading	Cronbach α
HSQ1	0.843	0.931
HSQ2	0.833	
HSQ3	0.877	
HSQ4	0.934	

#### Reliability

**Table:**

Items	Factor Loading	Cronbach α:
HSQ5	0.948	0.947
HSQ6	0.879	
HSQ7	0.905	
HSQ8	0.860	
HSQ9	0.827	

#### Responsiveness

**Table:**

Items	Factor Loading	Cronbach α
HSQ10	0.750	0.882
HSQ11	0.930	
HSQ12	0.907	
HSQ13	0.662	

#### Assurance

**Table:**

Items	Factor Loading	Cronbach α
HSQ14	0.910	0.938
HSQ15	0.930	
HSQ16	0.844	
HSQ17	0.853	

#### Empathy

**Table:**

Items	Factor Loading	Cronbach α
HSQ18	0.909	0.945
HSQ19	0.806	
HSQ20	0.939	
HSQ21	0.855	
HSQ22	0.888	

### Attitudes

**Table:**

Items	Factor Loading	Cronbach α
ATT1	0.824	0.960
ATT2	0.940	
ATT3	0.956	
ATT4	0.979	

### Healthcare image

**Table:**

Items	Factor Loading	Cronbach α
HCI1	0.849	0.923
HCI2	0.895	
HCI3	0.882	
HCI4	0.944	
HCI5	0.619	
HCI6	0.806	
